# Identifying malnutrition in emergency general surgery: systematic review

**DOI:** 10.1093/bjsopen/zrad086

**Published:** 2023-09-25

**Authors:** Daniel L Ashmore, Adil Rashid, Timothy R Wilson, Vanessa Halliday, Matthew J Lee

**Affiliations:** School of Medicine and Population Health, Faculty of Health, University of Sheffield, Sheffield, UK; Department of General Surgery, Doncaster and Bassetlaw Teaching Hospitals NHS Foundation Trust, Doncaster, UK; School of Medicine and Population Health, Faculty of Health, University of Sheffield, Sheffield, UK; Department of General Surgery, Doncaster and Bassetlaw Teaching Hospitals NHS Foundation Trust, Doncaster, UK; School of Medicine and Population Health, Faculty of Health, University of Sheffield, Sheffield, UK; School of Medicine and Population Health, Faculty of Health, University of Sheffield, Sheffield, UK

## Abstract

**Background:**

Emergency general surgery practice is high risk. Surgery is a key part of treatment, with resultant catabolic stress and frequent need for nutritional support. The aim of this study was to examine the current methods of defining and determining malnutrition in emergency general surgery. This included examining the use of nutrition screening and assessment tools and other measures of malnutrition.

**Methods:**

MEDLINE, Embase, Cumulative Index to Nursing and Allied Health Literature, trial registries, and relevant journals published between January 2000 and January 2022 were searched for studies of adult patients with any emergency general surgery diagnosis, managed conservatively or operatively, with an assessment of nutritional status. Mixed populations were included if more than 50 per cent of patients were emergency general surgery patients or emergency general surgery results could be separately extracted. Studies in which patients had received nutritional support were excluded. The protocol was registered with PROSPERO, the international prospective register of systematic reviews (CRD42021285897).

**Results:**

From 6700 studies screened, 324 full texts were retrieved and 31 were included in the analysis. A definition of malnutrition was provided in 23 studies (75 per cent), with nutritional status being determined by a variety of methods. A total of seven nutrition screening tools and a total of nine ‘assessment’ tools were reported. To define malnutrition, the most commonly used primary or secondary marker of nutritional status was BMI, followed by albumin level.

**Conclusion:**

Wide variation exists in approaches to identify malnutrition risk in emergency general surgery patients, using a range of tools and nutrition markers. Future studies should seek to standardize nutrition screening and assessment in the emergency general surgery setting as two discrete processes. This will permit better understanding of malnutrition risk in surgical patients.

## Teaser

There is a wide variation in approaches to identify malnutrition risk in emergency general surgery patients, using a range of tools and nutrition markers, with significant heterogeneity. Work is needed to standardize the approach to determining a patient’s nutritional status in the emergency setting.

## Introduction

Emergency general surgery (EGS) is the single largest group of surgical patients admitted to hospital in the UK^1^. EGS represents 4.2 per cent of all hospital admissions in the UK and 7.1 per cent in the USA^2^. More than a quarter of patients admitted in this setting require surgery^2,3^, which carries a high risk of morbidity and mortality^4–6^. Malnutrition is an established risk factor for worse outcomes, including more frequent nosocomial infections^7^, extended length of stay^6^, increased mortality^5^, and increased healthcare service costs^6,8,9^.

Despite the size of the challenge, there is no universally accepted definition for malnutrition in the literature or in EGS clinical guidelines^10–16^. The current incidence of malnutrition in EGS patients has not been clearly established, but may be as high as 40–60 per cent^5,17^. Knowing which patients are malnourished will allow targeted nutritional support, which may improve outcomes.

The identification of malnourished patients is typically a two-step process^18,19^. This involves an initial screening, followed by a more comprehensive assessment by an appropriate healthcare professional for those deemed to be at risk. There is an abundance of methods and tools to determine the risk of malnutrition in patients, though very few are validated in the EGS cohort. There is no international consensus on which screening tool should be used^12,15,20^.

The Malnutrition Universal Screening Tool (MUST) is one such screening tool that has been validated across multiple healthcare settings and populations. It was developed from a community tool by the Malnutrition Advisory Group of the British Association for Parenteral and Enteral Nutrition (BAPEN)^21,22^ and consists of three domains (body mass index (BMI), unintentional weight loss, and acute disease or lack of nutrition affect). A score of two or more out of six indicates a high risk of malnutrition and the need for specialist dietetic involvement. It is widely implemented across UK settings^23,24^ and is used in the majority of UK health institutions as the standard screening tool.

The aim of this study was to perform a systematic review of observational studies to describe how malnutrition is identified in EGS. It focused on nutrition screening and assessment tools, and their components, as well as other markers of malnutrition.

## Methods

### Protocol and registration

The systematic review was performed with reference to the Cochrane Handbook and reported using the PRISMA guidelines^25^. The protocol was registered with PROSPERO, the international prospective register of systematic reviews (CRD42021285897).

### Eligibility criteria

Studies of adult (aged 18 years and older) patients with any EGS diagnosis, managed conservatively or operatively, were included (*Table 1*). Participants must have had their nutritional status determined at some point in their hospital stay using a screening tool, assessment tool, or other measure of malnutrition. Studies on mixed patient populations (mixed surgical specialty, emergency and elective general surgical, or surgical and medical) were included if the EGS and non-EGS results were reported separately or if 50 per cent or more of the population reported were EGS patients. The same applied to studies of EGS and trauma patients. Both randomized and non-randomized studies were included, as the extracted data related to a descriptor, and were presented in a narrative manner. However, studies where a nutritional intervention implemented at any stage (oral, enteral, or parenteral) was described in the population were excluded, as observational studies were considered more likely to reflect real-world practice. Additional exclusions applied to studies of pregnant women, studies of patients with eating disorders, inappropriate study design (letters to the editor), review articles, and abstracts with no corresponding full text. Whilst studies published from any country were considered, non-English texts were excluded, as no resource was available to secure accurate and reliable translations.

**Table 1:** Inclusion/exclusion criteria

Included	Excluded
Adults (aged 18 years and older) EGS patients Any general surgical disease or operation Patients can be managed conservatively or operatively Must include an assessment of nutritional status (using a screening tool, assessment tool, or other measure) Published Jan 2000—Jan 2022 Any sex No geographical or healthcare facility restrictions	Non-general surgery patients Non-emergency patients (including studies of cancer resections where it is not clearly stated this was performed as an emergency) No assessment of nutritional status (using a nutritional screening tool, assessment tool, or other measure) Mixed study: studies on non-emergency patients (studies will be included if the EGS and non-EGS results are reported separately or if ≥50% of the population reported are EGS patients) Trauma study (studies will be included if the trauma and EGS results are reported separately or if ≥50% of the population reported are EGS patients) Patients received a nutritional intervention (including oral supplements and enteral or parenteral nutrition) at any stage (for example an RCT) Non-full texts, for example conference abstract only with no supporting full text Inappropriate study design (editorials) Reviews of the literature Non-English texts Pregnant women Eating disorders

EGS, emergency general surgery.

### Definitions

#### Nutrition screening tool

This was defined as a tool to identify individuals at risk of malnutrition. Although this would ordinarily subsequently lead to a full nutritional assessment by a dietitian, this was not necessary to be included in the review.

#### Nutrition assessment tool

Due to a lack of clear terminology in the literature, in this review a nutrition assessment tool was defined as a method that: characterized the nutritional status of an individual to confirm whether they were malnourished and/or to what degree; had ‘assessment’ in the name of the tool and was referred to by that throughout the study; or was used when the authors considered that a nutritional assessment (rather than screening) had taken place.

### Outcomes

The primary outcome of this review was to identify which nutrition screening or assessment tools are used in the EGS population and what their component parts are.

Secondary outcomes included mapping the range of alternative criteria used in defining malnutrition in EGS. This was purposefully kept broad to allow a full overview of the range of methods currently used to assess nutritional status. It included anthropometric indices (for example height, weight, BMI, and skin-fold thickness), biochemical markers (for example albumin and lymphocyte count), and others (for example wound healing and functional recovery).

### Search strategy

A systematic search for studies from January 2000 to January 2022 (sample search strategy available in the *Supplementary material*) was performed within the following databases: MEDLINE (via Ovid SP), Embase (via Ovid SP), Cumulative Index to Nursing and Allied Health Literature (CINAHL) (via EBSCO), and Cochrane CENTRAL. In addition, the following clinical trial registries were also searched: ClinicalTrials.gov, the WHO International Clinical Trials Registry Platform, and the EU Clinical Trials Register. Reference lists of eligible studies were checked, as were reports published by the relevant organizational bodies, including the Department of Health UK, the National Institute for Health and Care Excellence (NICE), the Royal College of Surgeons, and the British Society of Parenteral and Enteral Nutrition. Selected journals were searched, including *Clinical Nutrition*, the *Journal of Parenteral and Enteral Nutrition*, the *Journal of Human Nutrition and Dietetics*, *Nutrition and Dietetics*, and *BMC Nutrition*.

### Study selection and screening

Titles and abstracts were screened for eligibility by two independent reviewers (D.L.A. and A.R.) using Rayyan^26^. Manuscripts meeting eligibility, or where there was insufficient detail to make an assessment of eligibility, were obtained for full-text review. Disagreements, where necessary, were settled with a third reviewer (M.J.L.). All reasons for exclusion of studies were documented.

### Data extraction

A standardized data extraction sheet was used to extract details of relevant studies using Microsoft Excel (version 2010).

### Data items

The data to be extracted aimed to meet the outcomes as outlined above. This was performed by D.L.A. and validated by M.J.L. Disagreements were settled by majority decision between D.L.A, A.R., and M.J.L. Missing data were recorded as such. Duplicates were removed. Data items of interest included patient and disease characteristics, setting, and nutrition tools used. Markers of malnutrition were also identified.

### Data synthesis

A narrative synthesis was performed. This included reporting the frequency of nutrition tools used, the settings in which they were used, and the patient population included in the study. Included tools were reviewed to identify component items. These were mapped to ascertain ‘core areas’ of malnutrition used in the literature. Given this review reports a summary of how nutritional status is assessed rather than the clinical outcomes of patients, no bias assessment was performed for the studies included. This was not deemed necessary, in keeping with other similar reviews^27^.

## Results

### Search results

**Fig. 1:**
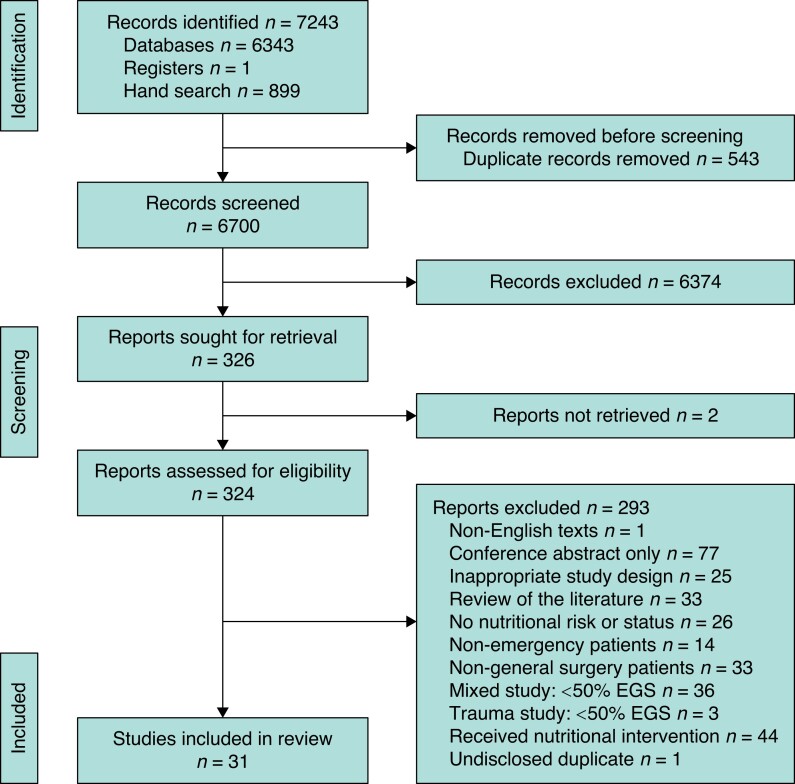
PRISMA flow diagram EGS, emergency general surgery.

The searches identified 7243 records, from which 543 duplicates were removed. Of the remaining 6700 studies screened for eligibility, 6374 studies were excluded based on title and abstract. A further two could not be retrieved. Full-text review of 324 studies was performed. The majority of these were conference abstracts with no corresponding full study (*Fig. 1*). There were 31 studies eligible for inclusion.

### Study characteristics

The summary characteristics of the included studies are detailed in *Table 2* (and *Table S1*). The aims of 14 studies (45.2 per cent) were specifically related to nutrition^5,28,30–41^. The majority of studies enrolled fewer than 100 EGS patients^30,36,37,40–46^. A total of seven studies included patients who all had a laparotomy^36,38,44–48^. A total of 15 studies were retrospective and a total of 15 studies were prospective (*Tables S2–S5*), with one study being unspecified in this respect (*Table S6*)^35^. Of the six (19.4 per cent) multicentre studies^31–34,39,49^, only three were prospective, with 1223 EGS patients (*Table S3*)^31,33,39^. EGS nutrition research was globally represented, with the majority of studies in Europe (11 studies (35.5 per cent)), followed by Asia (8 studies (25.8 per cent)) then North America (7 studies (22.6 per cent)). The vast majority of studies (19 studies (61.3 per cent)) did not disclose the hospital setting the study was performed in.

**Table 2:** Summary characteristics of the included studies

	Studies
**Year**	
2000–2005	3 (9.7)
2006–2010	2 (6.5)
2011–2015	6 (19.4)
2016–2020	14 (45.2)
2021–present day	6 (19.4)
**Type of study**	
Retrospective	15 (48.4)
Prospective	15 (48.4)
Not stated	1 (3.2)
**No. of centres**	
Single centre	25 (80.6)
Multicentre	6 (19.4)
**Setting**	
Africa	2 (6.5)
Asia	8 (25.8)
Australasia	2 (6.5)
Europe	11 (35.5)
North America	7 (22.6)
South America	1 (3.2)
**Hospital setting**	
Ward	4 (12.9)
ICU	2 (6.5)
Ward and ICU	6 (19.4)
Not stated/not applicable (follow-up study)	19 (61.3)*
**Number of EGS patients included in each study**	
<100	10 (32.3)
100–199	9 (29.0)
200–999	8 (25.8)
**≥**1000	3 (9.7)
**Proportion of patients in studies who were EGS patients**	
0–49%	3 (9.7)
50–99%	10 (32.3)
100%	18 (58.1)
**Mean patient age**	
<65 years	16 (51.6)
**≥**65 years	14 (45.2)
Not stated	1 (3.2)
**Criteria for malnutrition/at risk of malnutrition provided**	
Yes	23 (74.2)
No	8 (25.8)
**Timing of nutritional tool**	
At admission	8 (25.8)
Before surgery	7 (22.6)
After surgery	2 (6.5)
Before and after surgery	2 (6.5)
Not stated	12 (38.7)†

Values are *n* (%). A total of 31 studies were included. *One study is a follow-up study^28^. †One study stated day 4–6, but not clear if this was before or after surgery^29^. EGS, emergency general surgery.

### Definitions for malnutrition

Of the 31 studies included in this review, 23 studies (75 per cent) provided a definition of malnutrition. This was by use of a nutrition screening tool alone^33,34,39,48,50^, an assessment tool^28,29,32,37,38,40–42,51^, or a nutrition marker, either on its own^35,43,45^ or combined^5,31,44,49,52,53^. There were two studies that provided no definition of malnutrition^46,54^. In a further six studies the definition was not explicit due to an absence of laboratory value cut-offs^55^, an absence of scoring system cut-offs^56^, an absence of nutrition marker cut-offs^36,47,57^, or the tool used was for another reason (frailty) and malnutrition was not clearly defined within that tool or elsewhere in the study^58^. A total of three studies used a nutritional screening tool, followed by an assessment^5,30,48^, although the screening tool was not stated in one study^5^.

Overall, BMI was the most commonly used criterion to define malnutrition as either a primary or secondary marker of nutritional status, or as a key component of a nutritional tool, in 20 (64.5 per cent) studies. Serum albumin was used as the same in 18 (58.1 per cent) studies.

**Fig. 2:**
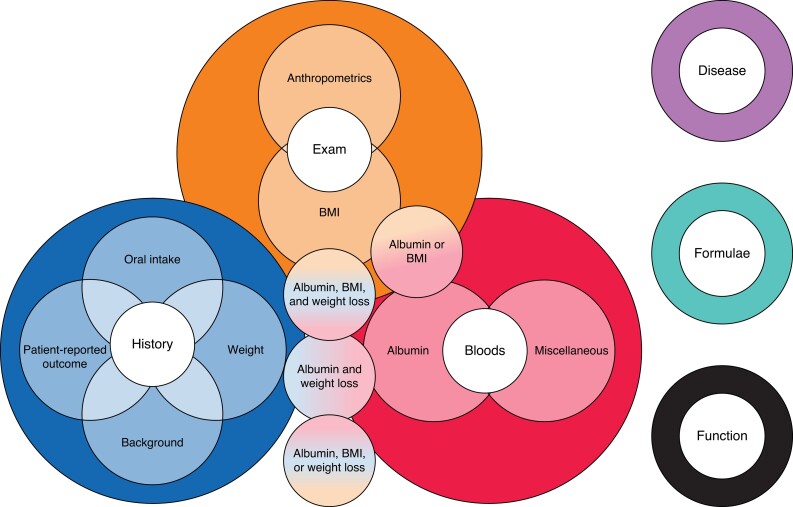
Mapping of criteria used to define malnutrition

The range of criteria for malnutrition according to a ‘core area’ is mapped in *Fig. 2*. These core areas are history, clinical examination, blood (laboratory) values, disease criteria, formulae, and functional tests. Core areas have underlying themes, which overlap with one another, representing the multiple criteria some studies used to define malnutrition (*Table S7*).

### Nutrition screening tools

Nutritional status was determined using a variety of methods, but each tool was used by one paper only (*Table 3*). There were seven nutrition screening tools or methods in use: the Hong Kong Chinese Malnutrition Screening Tool, the Malnutrition Screening Tool, hand-grip strength, the 3-Minute Nutrition Screening Tool, the Geriatric Nutritional Risk Index, the Trauma and Emergency General Surgery Frailty Index, and the Canadian Nutrition Screening Tool.

**Table 3:** Nutrition screening tools in emergency general surgery

Nutrition screening tool	Studies	Components	Score
Hong Kong Chinese Malnutrition Screening Tool (C-MUST)^*^	Ho *et al*.^33^, 2015	BMI Weight loss in last 3–6 months Acute disease precluding dietary intake for >5 days	Low risk = 0 Medium risk = 1 High risk = 2 Maximum = 6
Malnutrition Screening Tool (MST)	Byrnes *et al*.^29^, 2018	Weight loss Reduced oral intake	At risk of malnutrition = ≥2 Maximum = 5
Hand-grip strength (HGS)	Byrnes *et al*.^29^, 2018	Single Jamar hydraulic hand dynamometer (Lafayette Instrument, Lafayette, IN, USA) as per standardized positioning and instruction prescribed by the American Society of Hand Therapists (ASHT) and recorded as the mean of three trials (2–4-s isometric contraction with minimum of 30-s break in-between) in the dominant arm	Impaired = value below the lower limit of the 95% c.i. of the mean from age-, sex-, and side-specific normative data
3-Minute Nutrition Screening Tool (3-Min NST)	Chua and Chan^48^, 2020	Unintentional weight loss (past 6 months) Reduced nutritional intake (past week) Muscle wastage (from temple and clavicle)	Study criteria Malnutrition = ≥3 Original study criteria at risk of malnutrition = ≥3 Moderate malnutrition = 3–4 Severe malnutrition = 5–9
Geriatric Nutritional Risk Index (GNRI)	Jia *et al*.^34^, 2020	Formula based on the Buzby Index/NRI (1.489 albumin (g/l)) + (41.7 (weight/ideal weight)) Jia *et al*.^34^ also compared with albumin and BMI	Normal = >98 Mild malnutrition = ≥92–≤98 Moderate malnutrition = ≥82–<92 Severe malnutrition = ≥73–<82 Very severe malnutrition = <73
Trauma and Emergency General Surgery Frailty Index (TEGS FI)	Weiss *et al*.^50^, 2020	A composite tool with several categories designed to identify frailty: Co-morbidities Daily activities Health attitude Function Nutrition (albumin <3 g/dl)	Albumin <3 g/dl = 1 Albumin >3 g/dl = 0 Maximum = 15 TEGS frail = ≥4.875
Canadian Nutrition Screening Tool (CNST)†	Saravana-Bawan *et al*.^39^, 2021	Unintentional weight loss (past 6 months) Reduced nutritional intake (past week)	At risk of malnutrition = 2 ‘Yes’ answers

∗ The difference between C-MUST and the Malnutrition Universal Screening Tool (‘MUST’) is the lower cut-off values for BMI score. †CNST is based on MST, but the score is dichotomized (yes/no) rather than being based on points.

### Nutrition assessment tools

There were nine nutrition assessment tools, of which the most commonly used was the Subjective Global Assessment (SGA) tool. A summary of these tools is presented according to year published (*Table 4*). Nutrition screening and assessment tools according to ‘core areas’ are also presented (*Table 5*).

**Table 4:** Nutrition assessment tools in emergency general surgery

Nutrition assessment tool	Studies	Components	Score
Prognostic Nutrition Index (PNI)	Mohil *et al*.^38^, 2008; Takano *et al*.^40^, 2021	PNI (% risk) = 158–16.6 (Alb) – 0.78 (TSF) – 0.20 (TFN) – 5.8 (DH) Where Alb = albumin (g/dl); TSF = triceps skin-fold thickness (mm), TFN = serum transferrin (mg/dl), DH = skin test reactivity; delayed hypersensitivity to any of four recall antigens Original Buzby PNI index was used to identify patients at risk of postoperative complications	Low risk = <40% Intermediate risk = 40–49% High risk = ≥50% These match the original Buzby scoring criteria too
Subjective Global Assessment (SGA)	Koziel *et al*.^28^, 2017; Mohil *et al*.^38^, 2008; Chua and Chan^48^, 2020	A composite tool of patient history and a physical examination with a subjective rating of nutrition risk History: weight change (past 6 months and past 2 weeks); dietary intake; gastrointestinal symptoms; functional capacity; disease and metabolic demand; physical examination, including loss of subcutaneous muscle, muscle wasting, ankle oedema, sacral oedema or ascites	Well nourished = SGA A Moderate malnutrition = SGA B Severe malnutrition = SGA C
Patient-Generated Subjective Global Assessment (PG-SGA)	Daniele *et al*.^42^, 2015; Byrnes *et al*.^29^, 2018	As per SGA except that the medical history is completed by the patient themselves. A subjective classification of degree of risk by a healthcare professional remains	Well nourished = PG-SGA A Moderate malnutrition = PG-SGA B Severe malnutrition = PG-SGA C
Geriatric 8 (G8)	Kenig *et al*.^58^, 2015	The G8 score is composed of eight questions, seven based on MNA: Reduced intake past 3 months Involuntary weight loss Mobility Neuropsychological problems BMI Polypharmacy Self-assessment (global in comparison with others of similar age) Age	Frail ≤14 Non-frail >14 Maximum = 17
Nutrition Risk Index (NRI)/Buzby Index	Mambou Tebou *et al*.^37^, 2017	Formula used in study: NRI = 1.519 × serum albumin (g/l) + (0.417 × actual weight/ideal weight) Original Buzby Index: NRI = (15.9×serum albumin, g/l) + (0.417× % usual body weight) Where % usual body weight is current weight as a % of usual weight either 2 or 6 months ago^59^	Mild malnutrition = >97.5 Moderate malnutrition = 83.5–97.5 Severe malnutrition = <83.5
Mini Nutritional Assessment Short Form (MNA-SF)	Fuertes-Guiró *et al*.^51^, 2019	Based on MNA, this is a composite of six components: Reduced intake past 3 months Involuntary weight loss Mobility Psychological stress or acute disease in past 3 months Neuropsychological problems (dementia) BMI (or CC if unable to obtain BMI)	Normal = 12–14 At risk of malnutrition = 8–11 Malnourished = 0–7 Maximum = 14
Modified Glasgow Prognostic Score (mGPS)	Takano *et al*.^40^, 2021	CRP ≤10 mg/l + any albumin = 0 CRP >10 mg/l + albumin ≥3.5 g/dl = 1 CRP >10 mg/l + albumin <3.5 g/dl = 2	Malnutrition = 2
Mini-Nutritional Assessment (MNA) Full Form	Welch *et al*.^41^, 2021	A composite of 18 questions relating to: Anthropometric assessment (BMI, MAC, CC, weight loss past 3 months) General assessment (independent living, polypharmacy, psychological stress or acute disease in past 3 months, mobility, neuropsychological problems, pressure ulcers) Dietary assessment (number of daily meals, composition of meals, mode of feeding) Self-assessment (nutritional—do they think they have a nutritional problem, global in comparison with others of similar age)	Normal = ≥24 At risk of malnutrition = 17–23.5 Malnourished = <17 Maximum = 30
Modified Global Leadership on Malnutrition (mGLIM)	Haines *et al*.^32^, 2021	An adapted set of criteria in order to apply GLIM definitions to NSQIP data Phenotypic GLIM malnutrition criteria Low BMI (≤20 kg/m^2^ in patients aged ≤70 years and ≤22 kg/m^2^ in patients aged >70 years) Recent weight loss (>5% within the last 6 months or >10% beyond 6 months) Reduced muscle mass by a validated technique measured (not included) Aetiological GLIM malnutrition criteria Acute disease/injury represented by EGS patients (the NSQIP database) and a surrogate marker of inflammation (low admission albumin, ≤3.5 g/dl). A decreased albumin is a widely utilized NSQIP definition of preoperative malnutrition, but is not part of GLIM criteria Reduced food intake or assimilation (≤50% energy requirements for >1 week or any reduction for >2 weeks) or any gastrointestinal conditions that adversely affect food assimilation or absorption (original GLIM criteria not included in mGLIM)	All four criteria (mGLIM positive) = malnourished GLIM criteria require patients to be identified as at risk of malnutrition using a validated risk tool, and a subsequent diagnosis of malnutrition based on one phenotypic criterion and one aetiological criterion

Note: Sánchez Acedo *et al*.^56^ (2020) used MNA-SF, but no cut-off values were provided; only two criteria given (no malnutrition, malnourished) when MNA-SF has three (no malnutrition, at risk, malnourished). CC, calf circumference; CRP, C-reactive protein; MAC, mean arm circumference; NSQIP, National Surgical Quality Improvement Program; EGS, emergency general surgery.

**Table 5:** Nutrition screening and assessment tools according to ‘core area’

	Oral intake	Patient-reported outcome	Patient’s background	Weight	BMI	Anthropometrics	Albumin	Disease	Function
**Nutrition screening tools in EGS**									
Hong Kong Chinese Malnutrition Screening Tool (C-MUST)*	.	.	.	x	x	.	.	x	.
Malnutrition Screening Tool (MST)	x	.	.	x	.	.	.	.	.
Hand-grip strength (HGS)	.	.	.	.	.	.	.	.	x
3-Minute Nutrition Screening Tool (3-Min NST)	x	.	.	x	x	x		x	
Geriatric Nutritional Risk Index (GNRI)	.	.	.	x	.	.	x	.	.
Trauma and Emergency General Surgery Frailty Index (TEGS FI)	.	x	x	.	.	.	x	.	x
Canadian Nutrition Screening Tool (CNST)†	x	.	.	x		.	.	.	.
**Nutrition assessment tools in EGS**									
Prognostic Nutrition Index (PNI)	.	.	.	.	.	x	x	.	.
Subjective Global Assessment (SGA)	x	.	x	x	.	x	.	x	x
Patient-Generated Subjective Global Assessment (PG-SGA)	x	.	x	x	.	x	.	x	x
Geriatric 8 (G8)	x	x	x	x	x	.	.	.	x
Nutrition Risk Index (NRI)/Buzby Index	.	.	.	x	.	.	x	.	.
Mini Nutritional Assessment Short Form (MNA-SF)	x	.	x	x	x	x	.	x	x
Modified Glasgow Prognostic Score (mGPS)	.	.	.	.	.	.	x	.	.
Mini-Nutritional Assessment (MNA) Full Form	x	x	x	x	x	x	.	x	x
Modified Global Leadership on Malnutrition (mGLIM)	.	.	.	x	x	.	x	x	.

∗ The difference between C-MUST and the Malnutrition Universal Screening Tool (‘MUST’) is the lower cut-off values for BMI score. †CNST is based on MST, but the score is dichotomized (yes/no) rather than being based on points. EGS, emergency general surgery.

### Alternative nutrition markers (single measures)

There were seven single measures used to define malnutrition (*Table 6*): albumin, arm muscle circumference, BMI, cholinesterase, haemoglobin, neutrophil to lymphocyte ratio, and the total number of lymphocytes. Albumin^28,40,43,45,55^ and BMI^28,35,40,54,57^ were the most commonly used measures (used in five studies each).

**Table 6:** Alternative nutrition markers in emergency general surgery (single measures)

Nutrition measure	Studies	Criteria
Albumin	Farrah *et al*.^55^, 2013	Admission albumin (cut-off NS)
	Koziel *et al*.^28^, 2017; Fernandes *et al*.^43^, 2019	Malnutrition: albumin ≤3.5 g/dl
	Krishna *et al*.^45^, 2019	Preoperative albumin (reference value 3.5–5.5 g/dl) was used as an indicator of the nutritional status of the patients Significant hypoalbuminaemia (that is malnutrition) = 3.0 g/dl
	Takano *et al*.^40^, 2021	Preoperative levels (cut-off NS, although Table 3 suggests <3.6 g/dl)
Arm muscle circumference	Koziel *et al*.^28^, 2017	Anthropometric examinations (measurements of body weight, height, arms, waist, hip circumference, and skin-fold thickness) were used to calculate the BMI and arm muscle circumference
BMI	Khan *et al*.^35^, 2016	Malnourished: BMI <18.50 kg/m^2^ Properly nourished: BMI 18.50–24.99 kg/m^2^
	Koziel *et al*.^28^, 2017; Takano *et al*.^40^, 2021	Malnourished: BMI <20 kg/m^2^
	Küpper *et al*.^54^, 2015	Patients with a BMI >35 kg/m^2^ were included, although it was not clear that this constituted a definition of ‘malnutrition’
	Serejo *et al*.^57^, 2007	Dystrophic: BMI <21 or ≥30 kg/m^2^ Eutrophic: BMI ≥21 to <30 kg/m^2^ It was not clear if dystrophia constituted ‘malnutrition’
Cholinesterase	Takano *et al*.^40^, 2021	Preoperative levels; malnutrition: <199 U/l
Haemoglobin	Takano *et al*.^40^, 2021	Preoperative levels; NS, although Table 3 suggests <12.3 g/dl
Neutrophil to lymphocyte ratio	Takano *et al*.^40^, 2021	Preoperative levels; NS
TNL	Koziel *et al*.^28^, 2017	TNL <1200 in 1 mm^3^ of blood To determine the weakening of resistance that accompanies malnutrition, the TNL was determined by: TNL = (% of lymphocytes × number of lymphocytes)/100

NS, not specified; TNL, total number of lymphocytes.

### Alternative nutrition markers (combined measures)

There were seven combined measures used to define malnutrition between nine studies^5,31,34,36,44,47,49,52,53^. Hypoalbuminaemia was a key factor in eight of these. The combined measures included: appetite and weight loss; albumin and weight loss; albumin, BMI, or weight loss; albumin, BMI, and weight loss; albumin or BMI; a complex combined measure of weight loss, underweight based on ideal weight, muscle wasting, and inadequate protein-energy intake; and, BMI, arm circumference, and skin-fold thickness (*Table 7*).

**Table 7:** Alternative nutrition markers in emergency general surgery (combined measures)

Nutrition measure	Studies	Criteria
Appetite and weight loss	Barazanchi *et al*.^47^, 2020	Any weight loss in past 6 months and a recent reduction in appetite (reported by patients)
Albumin and weight loss	Mäkelä *et al*.^53^, 2005	Malnourished: albumin <35 g/l and weight loss in past 6 months
	Mäkelä *et al*.^52^, 2005	Malnourished if they had a serum albumin value <35 g/l and had experienced a weight loss of >5 kg during the past few months
Albumin, BMI, or weight loss	Kenig *et al*.^44^, 2012	Malnutrition: a decreased albumin level <30 g/l, or decreased body weight ≥10% or BMI <20.5 kg/m^2^
Albumin, BMI, and weight loss	Novy *et al*.^49^, 2021	Mild malnutrition (at least one of the following): BMI <21 kg/m^2^, weight loss 5% in 1 month or weight loss 10% in 6 months, albuminaemia <35 g/l Severe malnutrition (at least one of the following): BMI <18 kg/m^2^, weight loss 10% in 1 month, weight loss 15% in 6 months, albuminaemia <30 g/l
Albumin or BMI	Fentahun *et al*.^31^, 2021	Malnourished: BMI <18.5 or >24.9 kg/m^2^ Well nourished: BMI 18.5–24.9 kg/m^2^ Malnourished: albumin: <3.5 g/dl
	Jia *et al*.^34^, 2020	Malnourished: albumin <3.8 g/dl or BMI <18.5 kg/m^2^ (used to compare with GNRI)
Disease-related weight loss, underweight status based on % ideal body weight, muscle wasting, inadequate energy-protein intake Laboratory parameters are used in patients not considered to have inflammation or infection (exclusion criteria apply)	Havens *et al*.^5^, 2018	Severe protein-energy malnutrition (three of the following criteria must be met): Significant, disease-related, weight loss of >15% of usual weight within the past 6 months; <70% ideal body weight; pre-admission serum albumin <2.1 g/dl; TLC ≤800 mm^3^; transferrin <100 mg/dl; overt signs of muscle wasting on physical exam; inadequate energy intake (<50% of estimated needs for 3 days or <75% of estimated needs for 7 days) Moderate protein-energy malnutrition (two of the following criteria must be met): Significant, disease-related, weight loss of 10–15% of usual weight within the past 6 months; 70–84% of ideal body weight; pre-admission serum albumin 2.1–2.7 g/dl; TLC 800–1199 mm^3^; transferrin 100–149 mg/dl Mild protein-energy malnutrition (two of the following criteria must be met): Significant, disease-related, weight loss of 5–9% of usual weight within the past 6 months; 85–94% of ideal body weight; pre-admission serum albumin 2.8–3.4 g/dl; TLC 1200–1499 mm^3^; transferrin 150–199 mg/dl Non-specific protein-energy malnutrition: Known nutritional risk with metabolic stress and/or overt signs of malnutrition without supporting anthropometric or biochemical data. Clinical judgment is required to make this classification
BMI, MUAC, and skin-fold thickness	Lalhruaizela *et al*.^36^, 2020	No criteria given regarding MUAC or skin-fold thickness; however, BMI <18.5 kg/m^2^ was considered ‘low’ (not clear whether this meant malnourished)

GNRI, Geriatric Nutrition Risk Index; TLC, total leucocyte count; MUAC, mean upper arm circumference.

### Implementation of a nutritional tool

Patients’ nutritional status was assessed at a number of time points. Almost half of the studies (15 studies (48.4 per cent)) performed the assessment at admission or before surgery (*Table 2*).

## Discussion

This systematic review reports the current methods used to identify malnutrition in EGS. Within the 31 included studies, a range of approaches were used. These included seven nutrition screening tools, nine nutrition assessment tools, seven single nutrition markers, and seven combined measures used to define malnutrition. There is considerable heterogeneity in ‘core areas’ in the tools used in EGS research and their components.

The findings in this review likely reflect the inconsistency among guidelines and the absence of a universal definition for malnutrition. NICE suggests MUST to screen for malnutrition in the UK and this is widely adopted^12^. The European Society for Clinical Nutrition and Metabolism (ESPEN) recognizes a number of tools that can be used in a hospital setting. These include the Nutritional Risk Score 2002 (NRS 2002), the Mini Nutritional Assessment Short Form (MNA-SF) and MUST^15^. ESPEN’s guidelines on nutrition in critically ill patients advise that a general clinical assessment of nutrition should be performed until a specific tool has been validated, although every patient in an ICU for more than 48 h should be considered high risk for malnutrition^60^. Conversely, the Academy of Nutrition and Dietetics and the American Society for Parenteral and Enteral Nutrition (ASPEN) advise using nutritional risk screening tools such as NRS 2002 or Nutrition Risk in Critically Ill (NUTRIC)^20^. The Global Leadership Initiative on Malnutrition (GLIM) guidelines state that screening should be performed using a validated screening tool and, although none is recommended, tools such as NRS 2002, MNA-SF, MUST, and SGA are presented^14^.

The identification of malnutrition is a two-step process; screening of patients who may be at risk of malnutrition is followed by an assessment to confirm the diagnosis, categorize, and plan treatment. This is recognized by the British Dietetic Association^18^ and overseas by the Academy of Nutrition and Dietetics^19^, as well as a number of guidelines. The nutritional assessment is typically performed by a trained professional^18,19^. GLIM endorse that phenotypic (weight loss; BMI; muscle mass) and aetiological (food intake or assimilation; inflammation or disease burden) criteria can be used for a diagnosis of malnutrition, although consultation with a trained professional is recommended for a comprehensive assessment^14^. Only three studies used a nutritional screening tool, followed by an assessment^5,30,48^, although the screening tool was not stated in one study^5^. Of these three studies, the assessment of malnutrition was done by a dietitian in one study alone^5^. The absence of this two-step process in EGS research likely has two effects.

First, it leads to confusion within the literature, and possibly clinical practice, regarding what is meant by an ‘assessment’ of nutritional status. Some tools exist as nutrition ‘assessment’ tools in that a more global assessment is performed. Others are simply named assessment tools, but may be no more complex than a screening tool. Consequently, assessment tools are conflated with screening tools and vice versa. For example, SGA has been evaluated in many studies including of all types of adult inpatients^61^. It has subsequently become considered a gold standard against which other tools are assessed^61^. This is despite it being a subjective process with minimal ‘assessment’.

Second, the absence of a two-step process adds to the heterogeneity of EGS research, which is already a labyrinth of complex diagnostic, investigative, and management processes. This may further hamper the development of comparable studies and translation of malnutrition identification into routine practice. Of the nine ‘assessment’ tools in this review, three are based on formulae (Prognostic Nutrition Index (PNI), Nutrition Risk Index (NRI), and modified Glasgow Prognostic Score (mGPS)), two are subjective (SGA and Patient-Generated SGA (PG-SGA)) and three are aimed at identifying malnutrition within the framework of frailty (Mini Nutritional Assessment (MNA), MNA-SF, and Geriatric 8 (G8)).

Finally, albumin has often historically been regarded as a marker of nutritional status^62^ and there remains a degree of controversy with respect to its role in assessing malnutrition^63,64^. This study found that albumin was used in more than half of studies as a primary or secondary marker of malnutrition. It clearly has a role in the inflammatory state as a negative acute phase marker, the postoperative stress response, and determining disease severity; all of which may impact nutrition. Moreover, hypoalbuminaemia is associated with increased mortality and morbidity^65,66^ and waiting for this to normalize before surgery reduces these risks^67^. However, there is inadequate evidence for its direct role in determining nutritional status^68^. Indeed, it does not help in deciding which patients should receive nutritional support^69^ and a normal albumin level can be seen despite severe malnutrition^70^.

A number of international bodies advise against the use of albumin as a marker for malnutrition. NICE recommends that low albumin is more likely to be a reflection of acute disease rather than malnutrition^12^. ESPEN support this, but reiterate that hypoalbuminaemia is a surgical risk factor in its own right^60,71^. ASPEN categorically states that albumin and pre-albumin should not be used as nutrition markers^72^ and GLIM states that albumin is a proxy marker of inflammation only^14^. Although albumin may have a role in prognostication for surgical outcomes, the use of albumin as a marker of the current state of malnutrition should be abandoned, clinically and in future studies.

It is important to recognize, however, that a patient’s nutritional status is a dynamic process and, as such, so should be the process of identifying it. To begin with, there is the patient’s pre-existing nutritional status. They may be underweight, exhibit a degree of chronic malnutrition, or, ever-increasingly in the Western world, be overweight or obese. The subsequent treatment and management of their diagnosis, as well as future possibilities such as a persisting ileus or reduced oral intake, will also affect a patient’s nutritional status. Whilst almost half of the studies determined nutritional status at admission or before surgery, it was not stated in close to 40 per cent of studies and certainly many studies did not comment on whether patients were reassessed (data not stated).

This systematic review benefits from exploring a research question with direct relevance to a large proportion of acute surgical admissions. It was registered prospectively and had wide inclusion criteria, allowing for studies of adult patients with a general surgical diagnosis to be included, whether managed conservatively or operatively.

This study is not without limitations. Firstly, although more than half of the included studies (18 studies (58.1 per cent)) were confined to EGS patients, mixed patient populations (mixed surgical specialty, emergency and elective general surgical, surgical and medical, or trauma patients) were also eligible for inclusion, with the EGS group extracted. Although consideration was given to limit studies only to those of patients with ‘high-risk’ EGS diagnoses such as those defined by Symons *et al*.^73^ or those using National Emergency Laparotomy Audit (NELA) database criteria or similar^74^, this would have led to a paucity of data. A total of three studies had fewer than 50 per cent EGS patients^33,41,46^. The inclusion of these studies did not lead to irrelevant nutrition screening or assessment tools that were more targeted to populations other than EGS patients. Ho *et al*.^33^ was one of only three multicentre studies examining adult surgical patients undergoing elective and emergency operations on the luminal gastrointestinal tract. They used C-MUST, which is based on MUST and has been extensively validated and is widely used in the UK^21^. The second study, by Waqar *et al*.^46^, examined patients who had undergone a laparotomy in the elective, emergency, and trauma setting. In this study by Waqar *et al.*^46^, no definition of malnutrition is offered, although this was the case in a quarter of studies in this review. Lastly, the study by Welch *et al*.^41^, in which only 16 patients (19.8 per cent) were EGS patients, assessed the feasibility of conducting acute sarcopenia research. This study by Welch *et al.*^41^ used MNA. It is a well-validated tool for older populations and has questions relating to a range of anthropometric measures, including weight loss and BMI, and a dietary assessment^75^.

Finally, this review did not correlate how malnutrition was identified with the range of diagnoses, clinical outcomes, or patient-reported outcomes in EGS. This was not in the initial remit of the review, although it was noted that there were inconsistencies in reporting clinical outcomes. In addition to the various study designs, this would have been difficult to interpret and generate meaningful conclusions.

Given that there is a wide variation in study design, population, definitions, and the nutrition tools and markers used to determine malnutrition in EGS, caution must be used when interpreting the findings of these studies and applying them to the bedside. It is imperative to identify malnutrition in EGS, both at admission and throughout the duration of a patient’s hospital stay, but the current strategies are inadequate. Minimizing heterogeneity in study design and patient recruitment will be difficult in this setting given its very nature.

The literature identifies many nutrition screening tools and developing another new tool may not be helpful. It is crucial to emphasize screening and assessment as two distinct pathways. Gaining a better understanding as to how clinicians diagnose and manage malnutrition in EGS practice, and why they use the methods they do, may offer useful insights as to how this process can be made more robust and reproducible. It is recommended that future research in EGS populations should report malnutrition risk at baseline using a recognized tool to fully appreciate its impact on outcomes. Standardizing this would help with future data synthesis and interpretation.

Reducing variation will permit better understanding of malnutrition risk in surgical patients. Identifying patients who are malnourished, or at risk of being or becoming so, during their admission will allow targeted nutrition intervention and this may improve surgical outcomes.

## Supplementary Material

zrad086_Supplementary_Data

## Data Availability

The data that support the findings of this study are openly available in Figshare at http://doi.org/10.15131/shef.data.21710516.
