# Supplementary material for: Identifying malnutrition in emergency general surgery: systematic review
Source: BJS Open. 2023 Sep 25;7(5):zrad086. doi: 10.1093/bjsopen/zrad086 (PMC10519817; doi:10.1093/bjsopen/zrad086)
Supplement: zrad086_Supplementary_Data [file zrad086_supplementary_data.docx]

**Identifying Malnutrition in Emergency General Surgery (EGS): A Systematic Review**

Daniel L Ashmore^1,2^, Adil Rashid^1^, Timothy R Wilson^2^, Vanessa Halliday^3^, Matthew J Lee^1^

^1^Dept of Oncology and Metabolism, The Medical School, University of Sheffield, Sheffield, UK

^2^Doncaster and Bassetlaw Teaching Hospitals NHS Foundation Trust, Doncaster, UK

^3^School of Health and Related Research (ScHARR), University of Sheffield, Sheffield, UK

**Corresponding author:**

Mr Daniel L Ashmore (danielashmore@doctors.org.uk)^1^

^1^Dept of Oncology and Metabolism, The Medical School, University of Sheffield, Sheffield, UK, S10 2RX

ORCID ID 0000-0002-4321-5613; Twitter @realdanashmore

**Supplementary Materials - Index**

| **Supplementary Methods** |  |
| --- | --- |
| **Example search strategy for MEDLINE** | *Page 2* |
| **Supplementary Figures and Tables** |  |
| Supplementary Table 1: Summary characteristics of each included study | *Page 4* |
| Supplementary Table 2: A summary of single centre prospective studies | *Page 7* |
| Supplementary Table 3: A summary of multicentre prospective studies | *Page 11* |
| Supplementary Table 4: A summary of single centre retrospective studies | *Page 12* |
| Supplementary Table 5: A summary of multicentre retrospective studies | *Page 15* |
| Supplementary Table 6: A summary of a single centre study (not stated if retrospective or prospective) | *Page 16* |
| Supplementary Table 7: Criteria used in defining malnutrition according to ‘core areas’. | *Page 17* |

**Supplementary Methods**

**Example search strategy for MEDLINE**

1 exp Acute Disease/ (218603)

2 exp Emergencies/ (42306)

3 exp Emergency Service, Hospital/ (91083)

4 exp Surgery Department, Hospital/ (4557)

5 emergenc*.mp. (503997)

6 1 or 2 or 3 or 4 or 5 (720310)

7 exp General Surgery/ (40102)

8 exp Surgical Procedures, Operative/ (3369162)

9 General surg*.mp. (56033)

10 Surgery.mp. (2853383)

11 Abdom* surgery {Including Related Terms} (20903)

12 7 or 8 or 9 or 10 or 11 (4464105)

13 Laparotom*.mp. (62246)

14 (Celiotom* or coeliotom*).mp. (1369)

15 13 or 14 (63412)

16 Critical Care/ (57200)

17 Critical care {No Related Terms} (10095)

18 16 or 17 (58206)

19 12 or 15 or 18 (4523457)

20 6 and 19 (161313)

21 exp Nutrition Assessment/ (16651)

22 exp Nutritional Status/ (50027)

23 exp Malnutrition/ (129082)

24 Nutrition* risk {Including Related Terms} (7523)

25 Nutrition* assessment {Including Related Terms} (9443)

26 Nutrition* screening {Including Related Terms} (7198)

27 Nutrition index {Including Related Terms} (320)

28 Malnutrition {Including Related Terms} (10751)

29 Screening tool* {Including Related Terms} (52006)

30 Nutritional* deficienc* {Including Related Terms} (10751)

31 Malnutrition Screening Tool {Including Related Terms} (365)

32 MST {Including Related Terms} (6718)

33 Malnutrition Universal Screening Tool {Including Related Terms} (9611)

34 Global Leadership initiative on Malnutrition {Including Related Terms} (9262)

35 GLIM {Including Related Terms} (295)

36 Nutrition Risk Screening {Including Related Terms} (9314)

37 NRS {Including Related Terms} (3774)

38 Mini Nutritional Assessment {Including Related Terms} (7718)

39 MNA {Including Related Terms} (2528)

40 Mini Nutritional Assessment-Short Form {Including Related Terms} (9667)

41 MNA-SF {Including Related Terms} (425)

42 Subjective Global Assessment {Including Related Terms} (1693)

43 SGA {Including Related Terms} (9788)

44 Nutrition Risk in Critically ill {Including Related Terms} (7844)

45 NUTRIC {Including Related Terms} (96)

46 Modified Nutrition Risk in Critically ill {Including Related Terms} (7763)

47 mNUTRIC {Including Related Terms} (55)

48 Nutrition Risk Index {Including Related Terms} (7947)

49 NRI {Including Related Terms} (1912)

50 Geriatric Nutrition Risk Index {Including Related Terms} (46)

51 GNRI {Including Related Terms} (403)

52 Short nutrition assessment questionnaire {Including Related Terms} (9117)

53 SNAQ {Including Related Terms} (145)

54 21 or 22 or 23 or 24 or 25 or 26 or 27 or 28 or 29 or 30 or 31 or 32 or 33 or 34 or 35 or 36 or 37 or 38 or 39 or 40 or 41 or 42 or 43 or 44 or 45 or 46 or 47 or 48 or 49 or 50 or 51 or 52 or 53 (307047)

55 20 and 54 (1092)

56 limit 55 to (English language and yr="2000 -Current") (859)

Supplementary Table 1: Summary characteristics of each included study

| Author & Year | Hospital setting | Setting | Multicentre | Mean age, years (range) | EGS/ Total patients, % (n) | Laparotomy frequency (%) | Malnutrition defined | Nutrition measure studied* |
| --- | --- | --- | --- | --- | --- | --- | --- | --- |
| Barazanchi *et al.,* 2020^47^ | NS | Australasia | No | 62 (18-96) | 94.6 (717/758) | 100.0 | No | 6-month weight loss  Reduction in appetite |
| Byrnes *et al.,* 2018^29^ | Ward | Australasia | No | 74 (NS) | 58.7 (44/75) | NS | Yes | Screening: MST and HGS  Assessment: PG-SGA  *Weight, BMI* |
| Chua and Chan, 2020^48^ | Ward/ ICU | Asia | No | 60.5 (NS) | 100.0 (170/170) | 100.0 | Yes | Screening: (3-Min NS) Assessment: SGA  *BMI, albumin* |
| Daniele *et al.,* 2015^42^ | NS | Europe | No | 63 (24-79) | 55.0 (22/40) | NS | Yes | PG-SGA  *BMI, total serum protein, albumin, CRP* |
| Farrah *et al.,* 2013^55^ | Ward/ ICU | N. America | No | 58.9 (NS) | 100.0 (231/231) | NS | No | Albumin |
| Fentahun, Netsanet and Menber, 2021^31^ | NS | Africa | Yes | 39.7 (NS) | 51.3 (159/310) | 39.4 | Yes | BMI or albumin |
| Fernandes *et al.,* 2019^43^ | ICU | Europe | No | 64.5 (47–81) | 60.0 (36/60) | 68.3 | Yes | Albumin  *BMI* |
| Fuertes-Guiró, Vitali-Erion and Rodriguez Fernandez, 2019^51^ | Ward | Europe | No | 78.6 (NS) | 100.0 (286/286) | NS | Yes | MNA SF  *Albumin,*  *skeletal muscular mass (measured via bioimpedence)* |
| Haines *et al.,* 2021^32^ | NS | N. America | Yes | 61.4 (NS) | 100.0 (31029/31029) | NS | Yes | mGLIM |
| Havens *et al.,* 2018^5^ | ICU | N. America | No | 61 (NS) | 100.0 (1361/1361) | NS | Yes | Weight loss, ideal weight, muscle wasting, oedema, intake, clinical judgement, biochemical markers (albumin, TLC, transferrin) |
| Ho *et al.,* 2015^33^ | NS | Asia | Yes | 65.9 (19-95) | 40.3 (380/943) | NS | Yes | C-MUST |
| Jia *et al.,* 2020^34^ | NS | N. America | Yes | 75  (IQR 69-82) | 100.0 (82725/82725) | NS | Yes | GNRI, albumin or BMI |
| Kenig *et al.,* 2012^44^ | Ward/ ICU | Europe | No | 66.8 (NS) | 80.4 (45/56) | 100.0 | Yes | Albumin or weight loss or BMI |
| Kenig *et al.,* 2015^58^ | Ward | Europe | No | 76.9  (65-100) | 100.0 (184/184) | NS | No | G8  *Albumin* |
| Khan, Ahmed and Safdar, 2016^35^ | NS | Asia | No | NS (15-60) | 100.0 (200/200) | 20 | Yes | BMI |
| Mäkelä, Kiviniemi and Laitinen, 2005^54^ | NS | Europe | No | 81.5 (NS) | 100.0 (218/218) | NS | Yes | Albumin and weight loss  *BMI* |
| Mäkelä, Kiviniemi and Laitinen, 2005^53^ | NS | Europe | No | 65 (28-99) | 100.0 (172/172) | 95.9 | Yes | Albumin and weight loss  *BMI* |
| Koziel *et al.,* 2017^28^ | N/A | Europe | No | 54.4 (NS) | 100.0 (150/150) | NS | Yes | SGA, BMI, albumin, TNL, AMC |
| Krishna *et al.,* 2019^45^ | NS | Asia | No | 52 (NS) | 100.0 (30/30) | 100.0 | Yes | Albumin |
| Küpper *et al.,* 2015^52^ | Ward/ ICU | N. America | No | 53 (17–85) | 100.0 (111/111) | 35.1 | No | BMI |
| Lalhruaizela *et al.,* 2020^36^ | NS | Asia | No | 48.3 (19-70) | 68.7 (68/99) | 100.0 | No | MUAC, skin fold thickness and BMI |
| Mambou Tebou *et al.,* 2017^37^ | NS | Africa | No | 34.9 (19-50) | 67.1 (57/85) | NS | Yes | NRI/ Buzby index  *BMI, albumin* |
| Mohil *et al.,* 2008^38^ | NS | Asia | No | 33 (13-75) | 100.0 (101/101) | 100.0 | Yes | PNI or SGA |
| Novy *et al.,* 2021^49^ | Ward/ ICU | Europe | Yes | 83 (79-89) | 100.0 (180/180) | NS | Yes | BMI, weight loss and albumin |
| Sánchez Acedo *et al.,* 2020^56^ | NS | Europe | No | 78 (65-103) | 100.0 (446/446) | NS | No | MNA SF |
| Saravana-Bawan *et al.,* 2021^39^ | NS | N. America | Yes | 74.6 (NS) | 100.0 (684/684) | NS | Yes | CNST |
| Serejo *et al.,* 2007^57^ | NS | S. America | No | 36.5 (NS) | 100.0 (266/266) | NS | No | BMI |
| Takano *et al.,* 2021^40^ | NS | Asia | No | 80.1 (NS) | 100.0 (60/60) | NS | Yes | Cholinesterase  *PNI, mGPS, albumin* |
| Waqar *et al.,* 2005^46^ | NS | Asia | No | 39.7 (13-78) | 36.8 (43/117) | 100.0 | No | NS  *Obesity* |
| Weiss *et al.,* 2020^50^ | Ward/ ICU | N. America | No | 78.6 (NS) | 58.1 (137/236) | NS | Yes | Albumin (as part of Trauma and Emergency General Surgery Frailty Index (TEGS FI)) |
| Welch *et al.,* 2021^41^ | Ward | Europe | No | 79.2 (NS) | 19.8 (16/81) | 14.8 | Yes | MNA  *BMI* |

Abbreviations: 3-minute nutrition screening (3-Min NS); arm muscle circumference (AMC); body mass index (BMI); Canadian Nutrition Screening Tool (CNST); emergency general surgery (EGS); Geriatric 8 (G8); Geriatric Nutrition Risk Index (GNRI); hand grip strength (HGS); Hong Kong Chinese Malnutrition Universal Screening Tool (C-MUST); intensive care unit (ICU); Malnutrition Screening Tool (MST); mean upper arm circumference (MUAC); Mini Nutritional Assessment (MNA); Mini Nutritional Assessment short form (MNA SF); modified Glasgow prognostic score (mGPS); modified Global Leadership Initiative on Malnutrition (mGLIM); not applicable (N/A); not stated (NS); Nutrition Risk Index (NRI); Patient Generated Subjective Global Assessment (PG-SGA); Prognostic Nutrition Index (PNI); Subjective Global Assessment (SGA); total leucocyte count (TLC); total number of lymphocytes (TNL). *in addition to the main nutrition measure studied, other nutritional measures included in the study are italicised.

Supplementary Table 2: A summary of single centre prospective studies

| Author, year, setting | Inclusion and exclusion criteria | Mean age, years (range) | EGS patients, % (n EGS/ total) | Measurement tool & malnourished EGS patients, % (n) | Study aim | Primary outcome | Main nutrition outcome |
| --- | --- | --- | --- | --- | --- | --- | --- |
| Byrnes *et al.*, 2018^29^ | Inclusion: ≥65 years; length of stay ≥72 hours; not critically ill or palliated; can obtain informed consent  Exclusion: Unable to complete the test, or condition causing reduced hand function | 74 (NS) | 58.7 (44/75) | **EGS & elective: MST/ HGS**  MST: 32.0 (24/75) MST ≥2 and HGS: 13.3 (10/75)  **EGS only: PG-SGA**  PG-SGA B 18.2 (8/44)  PG-SGA C 2.3 (1/44) EGS Total 20.5 (9/44) | To determine discriminatory ability of HGS in screening for malnutrition as assessed by the PG-SGA and assess the added value of combining this with the MST. | HGS is not suitable in screening older inpatients for malnutrition during admission to surgical wards, nor does it increase the discriminatory ability of the MST. | As primary outcome |
| Fuertes-Guiró, Vitali-Erion and Rodriguez Fernandez, 2019^51^ | Inclusion: >65 years old diagnosed with urgent abdominal pathology and requiring immediate or urgent surgical treatment.   Exclusion: Inter- and intra-hospital ward transfers; previous elective surgery; transplants; unable to perform predictive tests; died during preoperative stabalisation | 78.6 (NS) | 100.0 (286/ 286) | **MNA SF**  Malnourished 29.4 (84/286) At Risk 31.6 (91/286) Normal 38.9 (111/286) | To assess frailty in a holistic context as a prognostic factor for the outcomes of geriatric patients undergoing emergency abdominal surgery, identifying the predictors that could be included in a global assessment score of preoperative frailty. | Frailty is a predictive factor that should be used routinely in geriatric EGS in a holistic context since no predictor is able to predict all the selected variables alone. The MNA SF, sarcopenia, Pfeiffer, Barthel and Duke tests best predict outcomes after emergency surgery. | There is an association of postoperative mortality and patients at risk of malnutrition (OR 1.89, p 0.032), in those who are malnourished (OR 1.57, p 0.14), low albumin (OR 2.94, p 0.038), low SMM (OR 2.78, p 0.022). Mortality 3 months after hospital discharge is associated with low SMM (OR 1.78, p 0.035). |
| Kenig *et al.*, 2015^58^ | Inclusion: ≥65 years needing emergency abdominal surgery  Exclusion: Unable to consent; immediate operation; incarcerated hernia with no laparotomy; operated >24 h after admission; abdominal wall infections; acute pancreatitis; patients managed endoscopically | 76.9 (65-100) | 100.0 (184/ 184) | **G8**  NS | To compare the diagnostic accuracy of six screening instruments in predicting frailty and postoperative morbidity and mortality. | The VES-13 was the best screening instrument; it had the highest sensitivity and negative predictive value both for the postoperative mortality and morbidity. | The G8 score was created based on the  MNA and the major cause of frailty is the  anorexia of aging, leading to protein energy malnutrition. Only 4 patients (11.4%) with fatal outcome were in good nutrition. |
| Koziel *et al.*, 2017^28^ | Inclusion: Severe acute pancreatitis (mild acute pancreatitis acted as control)  Exclusion: A moderate course of acute pancreatitis; chronic pancreatitis | 54.4 (NS) | 100.0 (150/ 150) | **SGA**  SAP  Normal 83.8 (85/99)  Malnourished 14.1 (14/99)  MAP  Normal 96.1 (49/51)  Malnourished 3.9 (2/51)  Additional results exist for BMI, albumin, TNL and AMC but these are not significant like SGA. | To evaluate pancreatic morphology and function, as well as nutritional status and quality of life among patients who experienced severe acute pancreatitis. | One year after SAP, patients exhibited changes in pancreatic morphology and carbohydrate metabolism disorders. Exocrine insufficiency equally among patients with severe or mild AP. The majority of QOL domains did not differ between patient groups. Patients with SAP described their mental health in more negative terms than patients with MAP (p<0.05). | A medium risk of malnutrition (SGA B) was observed in 16.2% (16) of patients after SAP vs. 2% (1) of patients after MAP (p=0.01).  No patients with SAP were classified as having SGA C vs one patient after MAP. |
| Krishna *et al.*, 2019^45^ | Inclusion: RL performed within 60 days of an initial laparotomy for complications arising following the primary surgery  Exclusion: Elective RL, index laparotomy performed in other hospital, damage control laparotomy, minimally invasive surgery | 52 (NS) | 100.0 (30/ 30) | **Pre-operative serum albumin levels**  53.3 (16/30) | To determine the incidence, indications, and outcome of RLs and identify factors affecting mortality and outcomes of RLs in Indian population. | RL was more common in emergency surgeries (80% vs 20%). The mean hospital stay was 25.8 days. The mean interval between first laparotomy and RL was 12.3 days and the interval between time of detection of the complication and RL was 3.96 days. | Mean serum albumin was 3.4 g/dL (range 2.2-4.3 g/dL). Mean albumin associated with mortality was 3.01. Five of seven patients (71%) who underwent RL for anastomotic dehiscence had hypoalbuminemia and four out of six patients (66%) undergoing RL for burst abdomen had hypoalbuminemia. |
| Lalhruaizela *et al.*, 2020^36^ | Inclusion: >18 years with pre-operative albumin level <3 g/dL; emergency and elective laparotomy for GI diseases  Exclusion: albumin ≥3.0 g/dL, clinically significant hypoalbuminemia; sepsis; jaundice; haemoglobin <8g/dL; PMH or FHx of T2DM/ CLD/ CKD/ hepatitis; protein-losing enteropathy/ nephrotic syndrome/ analbuminemia/ UC/ CF/ HF/ amyloidosis; taking steroids, hepatotoxic drugs, chemotherapy; BMI <12 or >40; alcohol or tobacco addiction; lost at follow-up | 48.3 (19-70) | 68.7 (68/99) | **BMI, MUAC and skin fold thickness**  Unknown as no criteria given | To determine the relationship between hypoalbuminemia (<3g/dL) and developing complications and mortality following laparotomy for GI diseases for both emergency and elective surgeries. | Pre-operative Hypoalbuminemia <3.0 gm/dL is a strong and independent risk factor for post-operative morbidity and mortality in GI surgeries and BMI is a strong indicator for post-operative complications. | The relationship of BMI and rate of complication was found to be statistically significant (p = 0.0001). Patients with BMI <18.5 kg/m^2^ had a complication rate of 89% (44/49). Patients with a BMI between 18.5-24.9 kg/m^2^ had a complication rate of was 26.7% (12/45) and 60% (3/5) between 25-30 kg/m^2^. |
| Mambou Tebou *et al.*, 2017^37^ | Inclusion: Consenting adults aged 19-59 years, either in the preoperative or postoperative period of abdominal surgery  Exclusion: Patients with GI malignancies | 34.9 (19-50) | 67.1 (57/85) | NS; figures for all patients  **NRI** Pre-op:  Moderately malnourished 30.4 (7/23)  Severely malnourished  8.7 (2/23)  Post-op:  Moderately malnourished 43.5 (37/85)  Severely malnourished 23.5 (20/85) | To assess the perioperative nutritional status of patients undergoing abdominal surgery and to determine the relationship between perioperative nutritional status and postoperative outcomes. | The prevalence of malnutrition is high and is associated with an increased risk of adverse early postoperative outcomes. Routine nutritional assessment and management is recommended. | The following were associated with a ‘normal evolution’: normal postoperative BMI [OR (95% CI) = 2.9 (1.19–7.34), p = 0.018]; normal postoperative albumin [OR (95% CI) = 55 (13.49–224.32), p < 0.001] and normal NRI [OR (95% CI) = 19.3 (2.18–169.79), p = 0.003]. |
| Mohil *et al.*, 2008^38^ | Inclusion: NS   Exclusion: Patients transferred postoperatively; uncomplicated acute appendicitis; early postoperative death; prolonged ventilatory support postoperatively, leaving hospital against medical advice | 33 (13-75) | 100.0 (101/ 101) | **PNI** Low risk 20.8 (21/101) Intermediate risk 14.9 (15/101) High risk 64.4 (65/101)  **SGA** SGA A 40.6 (41/101) SGA B 52.5 (53/101) SGA C 6.9 (7/101) | To study the association between nutritional status, morbidity, mortality and severity scores (POSSUM, P-POSSUM and APACHE II) in patients undergoing emergency laparotomy. | A majority of the patients had poor nutritional status on admission by both the nutritional assessment tools. With the onset of acute emergency, there was a significant relation of nutritional status with disease severity, morbidity and mortality. | SGA appears to be better assessing the pre-existing nutritional state. SGA was significantly associated with mortality, disease severity and overall morbidity (wound infection or dehiscence, anastomotic leak and chest infection). PNI significantly associated with wound dehiscence or infection and respiratory complications. |
| Sánchez Acedo *et al.*, 2020^56^ | Inclusion: aged ≥65 years who had undergone urgent abdominal surgery   Exclusion: NS | 78 (65-103) | 100.0 (446/ 446) | **MNA SF** No malnutrition 93.3 (415/445) Malnutrition 6.7 (30/445) | To analyse the incidence and factors associated with postoperative delirium in patients aged ≥65 who underwent urgent abdominal surgery. | Post-operative incidence of delirium was 13.6%, and was significantly associated with age, ASA III, physiological alteration in the ED, preoperative diagnosis, intestinal ischaemia and previous cognitive deterioration | Nutritional status (and comorbidity, frailty, functional dependence) were not statistically associated with delirium. Nourished and delirious patients 12.5% (52/415), whilst malnourished and delirious 26.7% (8/30). |
| Serejo *et al.*, 2007^57^ | Inclusion: Adults undergoing emergency abdominal surgery   Exclusion: diagnosis of NMD or acute respiratory condition on initial evaluation; extra-abdominal injuries; re-operations | 36.5 (NS) | 100.0 (266/ 266) | **BMI**  ‘Eutrophic’: ≥21 kg/m^2^ - <30 kg/m^2^) 77.1 (205/266)  ‘Dystrophic’: BMI <21 kg/m^2^ or ≥30 kg/m^2^ 22.9 (61/266) | The aim of this study was to determine the incidence and predictors of pulmonary complications following emergency abdominal surgery. | PPC developed in 75 patients (28.2%): 44 pneumonia, 16 atelectasis, 9 pleural effusion and 6 acute respiratory failure. ‘Dystrophic’ BMI, advanced age, type of incision were independent predictors of PPC. | Eutrophic: 24.9% (51/205) developed PPC. Crude OR = 1.95 (95% CI = 1.07–3.58; p value = 0.029)  Dystrophic 40% (24/61) developed PPC. |
| Waqar *et al.*, 2005^46^ | Inclusion: Adults undergoing laparotomy for various indications  Exclusion: NS | 39.7 (13-78) | 36.3 (43/117) | **Malnutrition measure NS**  Obesity 12.8 (15/117) Malnourished 19.7 (23/117) | To determine the frequency and risk factors of wound dehiscence/ burst abdomen in patients undergoing emergency and elective laparotomies through midline incisions | Incidence of wound dehiscence was 6.0% (7/117) and is statistically significant when comparing emergency vs elective surgery. | With respect to wound dehiscence, 13.3% (2/15) patients were obese and 13.0% (3/23) were malnourished. |
| Welch *et al.*, 2021^41^ | Inclusion: ≥70 years in three cohorts: elective colorectal surgery, emergency abdominal surgery, medical patients with infections  Exclusion: unable to understand English or mobilise prior to admission; life expectancy <30 days | 79.2 (NS) 75.5 (NS) EGS | 19.8 (16/81) | **MNA** Normal ≥24 40 (6/15) At risk 17-23.5 60 (9/15) Malnourished <17 0 (0/15) | To assess the feasibility of conducting acute sarcopenia research in complex populations of hospitalised older adults. | Recruitment rates were higher in elective surgery patients compared to EGS or medical patients. Drop-out rates were not affected by age or frailty of participants. Completion rates of ultrasound quadriceps were higher than other procedures. | As primary outcome |

Abbreviations: Arm muscle circumference (AMC); body mass index (BMI); chronic kidney disease (CKD); chronic liver disease (CLD); cystic fibrosis (CF); emergency department (ED); emergency general surgery (EGS); family history (FHx); Gastrointestinal (GI); Geriatric 8 (G8); hand grip strength (HGS); heart failure (HF); intensive care unit (ICU); Malnutrition Screening Tool (MST); Mini Nutritional Assessment short form (MNA SF); neuromuscular disease (NMD); not stated (NS); Nutrition Risk Index (NRI); odds ratio (OR); past medical history (PMH); Patient Generated Subjective Global Assessment (PG-SGA); Prognostic Nutrition Index (PNI); quality of life (QOL); relook laparotomy (RL); Severe acute pancreatitis (SAP); skeletal muscle mass (SMM); Subjective Global Assessment (SGA); total number of lymphocytes (TNL); type 2 diabetes mellitus (T2DM); ulcerative colitis (UC); Vulnerable Elderly Survey-13 (VES-13).

Supplementary Table 3: A summary of multicentre prospective studies

| Author, year | Inclusion and exclusion criteria | Mean age, years (range) | EGS patients, % (n EGS/ total) | Measurement tool & malnourished EGS patients, % (n) | Study aim | Primary outcome | Main nutrition outcome |
| --- | --- | --- | --- | --- | --- | --- | --- |
| Fentahun, Netsanet and Menber, 2021^31^ | Inclusion: ≥18 years undergoing abdominal surgery; a proportional systematic sampling technique was used based on the last four-month abdominal surgical operation reports  Exclusion: NS | 39.7 (NS) | 51.3 (159/310) | **BMI**  50.0 (155)  **Albumin**  44.8 (139) | To assess the association between nutritional status and wound healing progress | Nutritional status had a strong association with good wound healing outcomes | As primary outcome Well-nourished patients were 2x (BMI) and 1.6 (albumin) more likely to have better wound healing than malnourished patients. |
| Ho *et al.*, 2015^33^ | Inclusion: Chinese adult surgical patients undergoing elective and emergency operations on the luminal gastrointestinal tract; can give informed verbal consent  Exclusion: NS | 65.9 (19-95) | 40.3 (380/943) | **C-MUST** EGS NS  **EGS and non-EGS:** Low risk 67.1 (633/943) Medium risk 15.8 (149/943) High 17.1 (161/943) | To determine the preoperative malnutrition risk of patients undergoing major and ultramajor gastrointestinal operations using the C-MUST and to correlate the nutritional risk with surgical outcomes as captured by the surgical outcomes monitoring and improvement program | EGS results:  Length of stay 19.4 days  30-day mortality 6.8%  60-day mortality 11.1% | Note this is based on elective and EGS patients: increasing malnutrition risk was an independent predictor of length of stay, 30-day mortality, 60-day mortality and minor medical complicationss |
| Saravana-Bawan *et al.*, 2021^39^ | Inclusion: ≥ 65 years; index admission; postoperative acute abdominal surgery  Exclusion: elective surgery; palliative surgery; trauma surgery; non-abdominal EGS; nursing home residents; inter- and intra-hospital transfers | 74.6 (NS) | 100.0 (684/ 684) | **CNST** 6 week post-discharge at risk of malnutrition  Control site  Pre-EASE 61.9 (39/63)  Post-EASE 37.5 (42/112)*  Intervention site  Pre-EASE 35.8 (34/95)  Post-EASE 27.5 (28/102) | To assess post-discharge patient reported outcomes regarding nutrition and QOL following the introduction of an Acute Care for the Elderly (ACE) model of care in an EGS population (secondary outcomes of the Elder-friendly Approaches to the Surgical Environment (EASE) study) | The clinically and economically beneficial EASE interventions do not appear to compromise quality of life, risk for malnutrition, or patient satisfaction in the post-discharge period | There was a statistically significant improvement in malnutrition risk over time at control and intervention sites both pre- and post-EASE |

Abbreviations: Acute Care for the Elderly (ACE); body mass index (BMI); Canadian Nutrition Screening Tool (CNST); Elder-friendly Approaches to the Surgical Environment (EASE); emergency general surgery (EGS); Hong Kong Chinese Malnutrition Universal Screening Tool (C-MUST); not stated (NS). *study reports this as 52% (n = 42).

Supplementary Table 4: A summary of single centre retrospective studies

| Author, year | Inclusion and exclusion criteria | Mean age, years (range) | EGS patients, % (n EGS/ total) | Measurement tool & malnourished EGS patients, % (n) | Study aim | Primary outcome | Main nutrition outcome |
| --- | --- | --- | --- | --- | --- | --- | --- |
| Barazanchi *et al.*, 2020^47^ | Inclusion criteria: EGS during acute admission or reoperation following complications post- elective surgery  Exclusion criteria: Obstetric/ gynaecological, aortic/ iliac, cholecystectomies, appendectomies, and trauma  * Identical to UK NELA criteria | 62 (18-96) | 94.6 (717/758) | **Appetite and weight loss**  NS | To assess the most commonly used risk prediction tools for EL | NELA risk prediction tool to be most predictive of mortality after EL, when compared with the currently used risk prediction tools in EL (P-POSSUM, APACHE-II, ACS-NSQIP) | The NELA, along with the three other risk prediction tools, can potentially be improved through the addition of measures of patient nutritional status and frailty |
| Chua and Chan, 2020^48^ | Inclusion criteria: Patients undergoing a laparotomy (clinical decision)  Exclusion criteria: <18 years | 60.5 (NS) | 100.0 (170/ 170) | **3-Min NS**  cut off (≥3): 21.2 (33)  **SGA**  SGA A 60.0 (78)  SGA B 38.5 (50)  SGA C 1.5 (2) | Compare the outcomes of elderly patients undergoing EL against the outcomes of non-elderly patients | Elderly patients suffer worse morbidity (LOS, ICU LOS, post-op complications) and mortality following emergency laparotomy | Despite a trend for malnutrition, the global 3-MinNS score and global SGA did not differ significantly between elderly and non-elderly patients (some component parts did) |
| Daniele *et al.*, 2015^42^ | Inclusion criteria: Patients with recurrent ovarian cancer who experienced bowel obstruction  Exclusion criteria: Patients BO for other gynecological malignancies, non-epithelial or borderline ovarian cancer, BO for benign causes, previous or concurrent malignancy, concomitant recto-vaginal fistula | 63 (24-79) | 55.0 (22/40) | **PG-SGA**  NS | Describe and to compare medical and surgical approaches in the management (survival, QOL) of MBO in ovarian cancer | Surgery has to be considered in patients without serious contraindications; otherwise, a combination of opioids, anti-emetics, corticosteroids, and anti-secretory drugs is the mainstay in patients with poor general condition | Cachexia, low performance status, and poor nutritional status were significant predictors of worse survival after MBO, independently by the treatment |
| Farrah *et al.*, 2013^55^ | Inclusion criteria: Patients undergoing gastrointestinal resection with intestinal anastomoses   Exclusion criteria: trauma patients and patients who underwent primary enterotomy/ perforation repair, or had colostomy creation without creation of anastomosis | 58.9 (NS) | 100.0 (231/ 231) | **Albumin**  NS | Anastomotic failure rate | There was an anastomotic failure rate of 11.1%, significantly more so in the stapled (15%) versus the hand sewn group (6.1%) | Mean admission albumin was shown to be statistically significant in the anastomotic failure group (mean 3.1) versus the no complication group (mean 3.5) |
| Fernandes *et al.*, 2019^43^ | Inclusion criteria: Surgical complexity level 4 and 5 according to the L. R. Pasternak classification and severity criteria  Exclusion criteria: NS | 64.5 (47–81) | 60.0 (36/60) | **Albumin**  NS | To analyse the outcomes of pulmonary complications in patients post-abdominal surgery and admitted to ICU | PPCs occur within 48 hours in 60% of abdominal surgical patients that need ICU care | Patients with a pre-operative serum albumin ≤3.5g/dL had a 19-fold greater odds of mortality within 30 days |
| Havens *et al.*, 2018^5^ | Inclusion criteria: ≥18 years admitted to the ICU and had an EGS procedure performed within 48 hours of admission.  Exclusion criteria: No dietitian assessment at ICU admission, planned readmissions, transplantation, procedures related to pregnancy, and psychiatric issues | 61 (NS) | 100.0 (1361/ 1361) | **Disease-related weight loss, underweight status based on percent ideal body weight, muscle wasting, inadequate energy or protein intake**  Malnutrition absent 32.5 (442) Non-specific malnutrition 59.7 (812) Protein-energy malnutrition 7.9 (107) | To determine whether pre-existing malnutrition is associated with increased risk of 90-day mortality following ICU admission among EGS patients | Mortality in-hospital and at 90 days was 10.1% and 17.9%, respectively.  Compared to patients without malnutrition, nonspecific malnutrition and protein-energy malnutrition increased the odds of 90-day mortality by 1.5-fold and 3.1-fold, respectively | In critically ill patients who undergo EGS, malnutrition at ICU admission is predictive of adverse outcomes. In survivors of hospitalization, malnutrition at ICU admission is associated with increases in readmission and mortality |
| Kenig *et al.*, 2012^44^ | Inclusion criteria: Laparotomies complicated by postoperative wound dehiscence; matched to age (±2-3 years), gender, underlying disease and the type of surgery performed  Exclusion criteria: NS | 66.8 (NS) | 80.4 (45/56) | **BMI**  Wound dehiscence group 21.4 (12/56)  Control group 14.3 (24/168) | To identify the main risk factors for abdominal wound dehiscence in adults | The most important risk factor for wound dehiscence is surgical site infection | Malnutrition had slightly increased odds of wound dehiscence (OR 1.2) but this was not significant |
| Mäkelä, Kiviniemi and Laitinen, 2005^54^ | Inclusion criteria: >75 years with a diagnosis of cholecystitisis  Exclusion criteria: NS | 1. 1988-1992, 81 (NS)  2. 1998-2002, 82 (NS) | 100.0 (218/ 218) | **Albumin and weight loss**  NS | To examine whether a change in treatment strategies over time has affected operation rates, morbidity and mortality | No difference in morbidity (44% vs 48%) or mortality (8% vs 6%). The rate of conservative management was similar (37% vs 30%), whilst the operative rate was significantly higher in period 1 (60% vs 25%). ERCPs and percutaneous cholecystostomy decreased the need for operations during period 2. | Malnutrition is an independent factor that contributes to mortality |
| Mäkelä, Kiviniemi and Laitinen, 2005^53^ | Inclusion criteria: Patients with perforated diverticulitis   Exclusion criteria: NS | 65 (28-99) | 100.0 (172/ 172) | **Albumin and weight loss**  9.3 (16) | Report of 20 years’ experience of acute perforated diverticulitis | Postoperative mortality was related to age, but age is not an independent prognostic factor for morbidity or mortality among elderly patients with perforated diverticular disease. | Malnutrition is a significant risk factor for mortality in patients aged over 70 years (0.014), but not quite in under 70 years (0.052). The patients who had experienced malnutrition preoperatively (p = 0.009) stayed longer in hospital. In total, 16/172 patients were malnourished. |
| Küpper *et al.*, 2015^52^ | Inclusion criteria: >17 years undergoing EGS with a BMI ≥35  Exclusion criteria: Elective surgery or BMI <35 | 53 (17–85) | 100.0 (111/ 111) | **BMI**  100.0 (111) | To assess perioperative outcomes in obese patients undergoing EGS | Obese patients requiring EGS are high risk for morbidity and mortality perioperatively Postoperatively, 40% (44) patients required ICU, 31% (34) required reoperation and 42% (47) had complications | There was no difference between severely obese and morbidly obese patients with respect to demographics, co-morbidities, operative factors, ICU LOS, severity of complications or mortality. After adjustment, neither a BMI of ≥40 or incremental increases in BMI conferred a significant effect on in-hospital mortality |
| Takano *et al.*, 2021^40^ | Inclusion criteria: Aged ≥65 years and undergoing major EGS   Exclusion criteria: Patients with acute appendicitis, incarcerated hernia or acute cholecystitis, in-hospital onset, re-operations, insufficient data | 80.1 (65-95) | 100.0 (60/ 60) | **Cholinesterase**  NS | To evaluate the predictive value of preoperative serum ChE levels as a risk factor for postoperative complications in the elderly who have undergone EGS | The preoperative serum ChE level was found to be an independent risk factor for elderly patients in emergency surgery | Preoperative serum ChE level may help to classify elderly patients according to their nutritional status and predict the postoperative course. ChE levels and PNI were statistically significant when comparing patients with a postoperative complication Clavien-Dindo ≥ grade II or not |
| Weiss *et al.*, 2020^50^ | Inclusion criteria: Patients admitted to the trauma and emergency general surgery service and were ≥65 years old  Exclusion criteria: NS | 78.6 (NS) | 58.1 (137/236) | **TEGS FI**  NS | To determine how the TEGS frailty index compares to an EMR-based frailty assessment in predicting geriatric TEGS outcomes | Multidimensional bedside frailty assessment tools were similar to comorbidity-focused, EMR-based frailty assessment tools in identifying geriatric TEGS patients at increased risk for complications and prolonged LOS | NS |

Abbreviations: 3-minute nutrition screening (3-Min NS); Acute Physiology and Chronic Health Evaluation-II (APACHE-II); American College of Surgeons National Surgical Quality Improvement Program (ACS NSQIP); bowel obstruction (BO); electronic medical record (EMR); emergency laparotomy (EL); intensive care unit (ICU); length of stay (LOS); malignant bowel obstruction (MBO); National Emergency Laparotomy Audit (NELA); not stated (NS); odds ratio (OR); Portsmouth-Physiological and Operative Severity Score for the enumeration of Mortality and morbidity (P-POSSUM); postoperative pulmonary complication (PPC); quality of life (QOL); Subjective Global Assessment (SGA); trauma and emergency surgery (TEGS) frailty index (FI) (TEGS FI); United Kingdom (UK). The Pasternak classification categorises highly invasive procedures, duration of surgery and intraoperative complications along with the usual postoperative PICU stay with invasive monitoring.

Supplementary Table 5: A summary of multicentre retrospective studies

| Author, year | Inclusion and exclusion criteria | Mean age, years (range) | EGS patients, % (n EGS/ total) | Measurement tool & malnourished EGS patients, % (n) | Study aim | Primary outcome | Main nutrition outcome |
| --- | --- | --- | --- | --- | --- | --- | --- |
| Haines *et al.*, 2021^32^ | Inclusion criteria: EGS patients enrolled in the NSQIP database (emergent gastrointestinal surgery on small bowel, colon, and rectum)  Exclusion criteria: Patients with missing albumin data or whose levels were below 0.1 g/dL | 61.4 (NS) | 100.0 (31029/ 31029) | **mGLIM**  1.7 (517) | To assess the post-operative outcomes (mortality, LOS and post-operative complications) of patients meeting a modified  GLIM-defined (mGLIM) malnutrition criteria undergoing EGS in the NSQIP database | Mortality: Colon 2.01 [1.55-2.61] (p = <0.001); small bowel 1.70 [1.08-2.67] (p = 0.022)  LOS: Colon: 3.13 [1.93-4.33] (p = <0.001); small bowel: 1.25 [0.43-2.94] (p = 0.145)  Post-operative complications: Colon 2.06 [1.61-2.62]; small bowel 2.30 [1.57-3.37] (p<0.001 for both) | Malnutrition is associated with poor clinical outcomes following EGS affecting LOS and mortality |
| Jia *et al.*, 2020^34^ | Inclusion criteria: EGS patients enrolled in the NSQIP database (patients aged ≥65years who underwent EGS no later than 12 hours after admission or after the onset of related symptomatology  Exclusion criteria: Patients with missing height, weight, or preoperative albumin | 75 (IQR 69-82) | 100.0 (82725/ 82725) | **GNRI**  Overall: 66.74 (55214/82725)  Mild: 18.3 (15,164/82725)  Moderate: 22.5 (18851/82725)  Severe: 15.4 (12699/82725)  Very severe: 10.6 (8770/82725)  **Albumin**  Overall: 65.6 (54,259/82725)  Mild 15.6 (12,947/82725)  Moderate 19.5 (16118/82725)  Severe 15.0 (12420/82725)  Very severe 15.4 (12774/82725)  **BMI**  4.9 (4091/82725) | To determine whether preoperative malnutrition, as measured by GNRI, is independently correlated with postoperative mortality and morbidity in the elderly ES patient. To compare the performance of GNRI to BMI and albumin | Frequency (%) of 30-day mortality in relation to:  **GNRI**  Mild: 9.3 (1404/15164)  Moderate: 15.5 (2873/18851)  Severe: 24.1 (3064/12699)  Very severe: 38.1 (3340/8770)  **Albumin (<3.8 g/dL)**  Mild: 9.0 (1167/12947)  Moderate: 14.3 (2298/16118)  Severe: 21.3 (2645/12420)  Very severe: 34.5 (4404/12774)  **BMI <18.5**  24.1 (984/4091) | As malnutrition worsened from mild to very severe, the risk of mortality, morbidity (results in paper), and hospital length of stay progressively increased |
| Novy *et al.*, 2021^49^ | Inclusion criteria: ≥75 years with severe acute cholangitis admitted to ICU  Exclusion criteria: NS | 83 (79-89) | 100.0 (180/ 180) | **Albumin, BMI and weight loss**  ICU patients (n = 85)  No malnutrition 21.2 (18/85) Mild malnutrition 14.1 (12/85)  Severe malnutrition 65.7 (55/85)  General ward patients (n = 95) No malnutrition 48.4 (46/95) Mild malnutrition 18.9 (18/95) Severe malnutrition 32.6 (31/95) | To describe the 6-month mortality in patients with severe acute cholangitis ≥75 years admitted to ICU (in comparison to the ward) and to identify factors associated with this mortality. | ICU  In-hospital: 17.6 (15/85)  3-months: 38.8 (33/85)  6-months: 48.2 (41/85)  Ward  In-hospital: 15.8 (15/95)  3-months: 16.8 (16/95)  6-months: 30.5 (29/95) | Malnutrition was statistically associated with six month mortality in ICU and ward patients |

Abbreviations: Body mass index (BMI); emergency general surgery (EGS); Geriatric Nutrition Risk Index (GNRI); Global Leadership Initiative on Malnutrition (mGLIM); intensive care unit (ICU); length of stay (LOS); National Surgical Quality Improvement Program (NSQIP); not stated (NS).

Supplementary Table 6: A summary of a single centre study (not stated if retrospective or prospective)

| Author, year | Inclusion and exclusion criteria | Mean age, years (range) | EGS patients, % (n EGS/ total) | Measurement tool & malnourished EGS patients, % (n) | Study aim | Primary outcome | Main nutrition outcome |
| --- | --- | --- | --- | --- | --- | --- | --- |
| (Khan, Ahmed and Safdar, 2016^35^ | Case-control study  Inclusion criteria: aged 15-60 years with acute appendicitis, obstructive hernia, peritonitis of less than 2 days duration and blunt or penetrating soft tissue injuries; requiring surgical procedure within 12 hours of admission, ASA-1 and ASA-II  Exclusion criteria: ASA ≥III, diabetes mellitus, chronic debilitation disease (chronic hepatitis B and C); immune-compromised | NS (15-60) | 100.0 (200/200) | **BMI**  Nourished (BMI 18.50-24.99)  50 (100)  Malnourished (BMI <18.50)  50 (100) | To determine the effects of pre-existing malnutrition on the overall outcome of surgery in emergency situation (chest infection and wound infection) | In malnourished patients, 27% had chest infections and 29% had wound infection (both significant). Whilst in nourished patients, 14% had chest infection and 18% had wound infections (not significant). | Preoperative malnutrition increases the morbidity rate |

Abbreviations: Body mass index (BMI); American Society of Anesthesiology (ASA); not stated (NS).

Supplementary Table 7: Criteria used in defining malnutrition according to ‘core areas’.

| Oral intake |
| --- |
| - Reduced oral intake (MST): intake past 1 week (3-Min NST, CNST); intake past 3 months ( MNA SF, G8)  - Dietary intake (SGA)  - Number of daily meals (MNA)  - Composition of meals (MNA)  - Mode of feeding (MNA) |
| Appetite and weight loss |
| Any weight loss in past 6-months and a recent reduction in appetite (reported by patients) |
| Patient reported outcome |
| - Health attitude (TEGS FI)  - Self-assessment: in comparison to others of similar age (G8); do they think they have a nutritional problem (MNA) |
| Background |
| - Age (G8)  - Co-morbidities (TEGS FI)  - Independent living (MNA)  - Gastrointestinal symptoms (SGA)  - Neuropsychological problems (G8, MNA SF, MNA)  - Polypharmacy (G8, MNA)  - Pressure ulcers (MNA) |
| Weight |
| - Weight loss (MST): in last 3-6 months (C-MUST); past 3 months (MNA); >5% within the last 6 months or >10% beyond 6 months (mGLIM)  - Involuntary weight loss (MNA SF, G8)  - Unintentional weight loss: past 6 months (3-Min NST); past 6 months (CNST)  - Weight change (past 6 months + past 2 weeks) (SGA) |
| Body mass index |
| Score points if:  - <18.5 one study and (C-MUST)  - <20 two studies and (3-Min NST)  - ≤20 in patients aged ≤70 years and BMI ≤22 in patients aged >70 (mGLIM)  - <23 (G8, MNA SF, MNA)  - <21 or ≥30 one study  - >35 one study |
| Anthropometrics |
| Loss of subcutaneous muscle (SGA)  - Muscle wasting (3-Min NST, SGA)  - Ankle oedema (SGA)  - Sacral oedema or ascites (SGA)  - Mean arm circumference (MNA)  - Calf circumference (MNA) |
| Albumin |
| - Albumin levels: <3.0g/dL (TEGS FI); ≤3.5g/dL (mGLIM)  - Admission levels, not stated  - Preoperative albumin: <3.0g/dL; Levels not stated |
| Miscellaneous |
| - CRP >10/L + albumin <3.5g/dL (mGPS)  - Cholinesterase levels <199U/L  - Haemoglobin, not stated  - Neutrophil to lymphocyte ratio, not stated  - Total number of lymphocytes, <1,200/ 1 mm^3^ |
| Disease… |
| *- precluding* intake for >5 days (C-MUST)  *- with* nutrition risk (3-Min NST)  *- and* metabolic demand (SGA)  *- or* psychological stress in past 3 months (MNA SF, MNA)  *- represented* by EGS patients (mGLIM) |
| Formulae |
| - **GNRI** (1.489 albumin [g/L]) + (41.7 [weight/ideal weight])  - **PNI** (%risk) = 158 – 16.6 (Alb) – 0.78 (TSF) – 0.20 (TFN) – 5.8 (DH)  - **NRI** (1.519 × serum albumin (g/L) + (0.417 × actual weight/ideal weight) |
| Function |
| - Handgrip strength  - Function (TEGS FI)  - Functional capacity (SGA)  - Mobility (MNA, MNA SF, G8)  - Daily activities (TEGS FI) |
| Albumin and weight loss |
| Malnutrition if:  - Albumin <35g/L and weight loss in past 6 months  - Albumin <35 g/L and weight loss >5Kg during past few months |
| Albumin, BMI and weight loss |
| Malnutrition if:  - BMI <21  - weight loss of 5% in 1 month or 10% in 6 months  - Albumin <35g/L |
| Albumin, BMI or weight loss |
| Malnourished if:  - Albumin <30g/L  - weight loss ≥10%  - BMI <20.5 |
| Albumin or BMI |
| Malnourished if:  - Albumin <3.5g/dL, BMI <18.5 or >24.9  - Albumin <3.8g/dL, BMI <18.5 |

Criteria used in defining malnutrition according to ‘core areas’.
